# Dichloridobis[(*S*)-2-hydroxy­propion­amide-κ^2^ *O*,*O*′]manganese(II)

**DOI:** 10.1107/S1600536808004066

**Published:** 2008-02-13

**Authors:** Pascale Lemoine, Bernard Viossat, Jean Daniel Brion, Alain Bekaert

**Affiliations:** a Laboratoire de Cristallographie et RMN Biologiques, UMR 8015 CNRS, Faculté des Sciences Pharmaceutiques et Biologiques de Paris Descartes, 4 Avenue de l’Observatoire, 75270 Paris Cedex 06, France; b Université de Paris XI, Faculté des Sciences Pharmaceutiques et Biologiques, Laboratoire de Chimie Thérapeutique BioCIS, UPRES-A 8076 CNRS, 5 Rue J. B. Clément, 92296 Châtenay-Malabry Cedex, France

## Abstract

In the title compound, [MnCl_2_(C_3_H_7_NO_2_)_2_], the Mn^II^ ion is bound to two Cl atoms and to four O atoms from two lacta­mide mol­ecules which act as bidentate ligands, giving rise to a highly distorted octa­hedral coordination geometry. The axial positions are occupied by one Cl atom and one O (hydr­oxy) atom. The values of the *cis* bond angles at the Mn atom are in the range 72.33 (5)–100.17 (6)°. Of the two possible coordination modes (*N*,*O*- or *O*,*O*-bidentate) in metal complexes with lacta­mide or its derivatives described in the literature, the title compound exhibits the *O*,*O*-bidentate mode. In the crystal structure, monomeric manganese(II) complexes are linked by several N—H⋯Cl, O—H⋯Cl and O—H⋯O hydrogen bonds, generating a three-dimensional network.

## Related literature

For related literature, see: Bekaert *et al.* (2005 ▶, 2007 ▶); Chen *et al.* (2006 ▶); Girma *et al.* (2005 ▶).
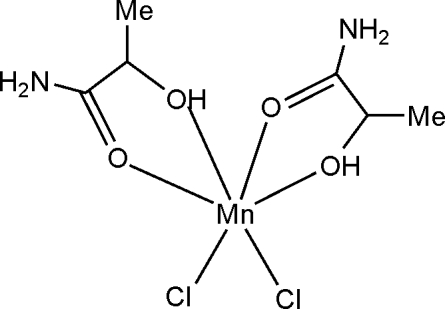


## Experimental

### 

#### Crystal data

[MnCl_2_(C_3_H_7_NO_2_)_2_]
*M* *_r_* = 304.03
Monoclinic, 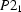

*a* = 6.312 (2) Å
*b* = 11.718 (3) Å
*c* = 8.268 (2) Å
β = 99.47 (1)°
*V* = 603.2 (3) Å^3^
*Z* = 2
Mo *K*α radiation
μ = 1.53 mm^−1^
*T* = 293 (2) K
0.18 × 0.16 × 0.12 mm

#### Data collection

Enraf–Nonius CAD-4 diffractometer
Absorption correction: none
3659 measured reflections
1836 independent reflections
1803 reflections with *I* > 2σ(*I*)
*R*_int_ = 0.030
3 standard reflections frequency: 60 min intensity decay: none

#### Refinement

*R*[*F* ^2^ > 2σ(*F* ^2^)] = 0.022
*wR*(*F* ^2^) = 0.061
*S* = 1.11
1836 reflections
161 parameters
1 restraint
H atoms treated by a mixture of independent and constrained refinement
Δρ_max_ = 0.34 e Å^−3^
Δρ_min_ = −0.51 e Å^−3^

### 

Data collection: *CAD-4 EXPRESS* (Enraf–Nonius, 1994 ▶); cell refinement: *CAD-4 EXPRESS*; data reduction: *XCAD4* (Harms & Wocadlo, 1995 ▶); program(s) used to solve structure: *SIR92* (Altomare *et al.*, 1994 ▶); program(s) used to refine structure: *SHELXL97* (Sheldrick, 2008 ▶); molecular graphics: *CAMERON* (Watkin *et al.*, 1996 ▶); software used to prepare material for publication: *WinGX* (Farrugia, 1999 ▶).

## Supplementary Material

Crystal structure: contains datablocks global, I. DOI: 10.1107/S1600536808004066/im2054sup1.cif

Structure factors: contains datablocks I. DOI: 10.1107/S1600536808004066/im2054Isup2.hkl

Additional supplementary materials:  crystallographic information; 3D view; checkCIF report

## Figures and Tables

**Table 1:** Hydrogen-bond geometry (Å, °)

*D*—H⋯*A*	*D*—H	H⋯*A*	*D*⋯*A*	*D*—H⋯*A*
N1—H1*A*⋯Cl1^i^	0.92 (4)	2.40 (4)	3.310 (2)	170 (3)
N1—H1*B*⋯Cl2^ii^	0.80 (3)	2.60 (4)	3.360 (2)	159 (3)
O2—H2⋯Cl1^iii^	0.89 (4)	2.26 (4)	3.1250 (17)	164 (3)
O7—H7⋯O1^iv^	0.86 (4)	1.83 (4)	2.676 (2)	167 (3)
N6—H6*A*⋯Cl2^v^	0.80 (4)	2.65 (4)	3.439 (2)	168 (3)
N6—H6*B*⋯Cl2^vi^	0.87 (4)	2.54 (4)	3.409 (3)	172 (4)

Symmetry codes: (i) 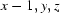; (ii) 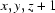; (iii) 
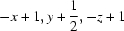
; (iv) 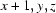; (v) 
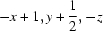
; (vi) 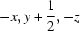.

**Fig. 1.**
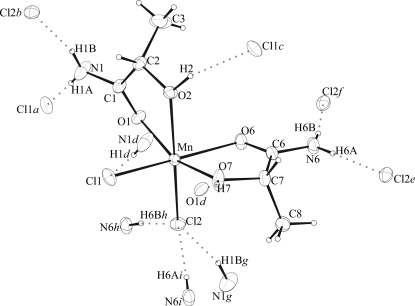
Molecular view of the complex showing the atomic numbering and some N—H···Cl, O—H···Cl and O—H···O hydrogen bonds as dotted lines. Displacement ellipsoids are displayed at the 50% probability level. Symmetry code: a: (x - 1, y, z), b: (x, y, 1 + z), c: (1 - x, 1/2 + y, 1 - z), d: (1 + x, y, z), e: (1 - x, 1/2 + y, -z), f: (-x, 1/2 + y, -z), g: (x, y, z - 1), h: (-x, y - 1/2, -z) and i: (1 - x, y - 1/2, -z).

**Fig. 2.**
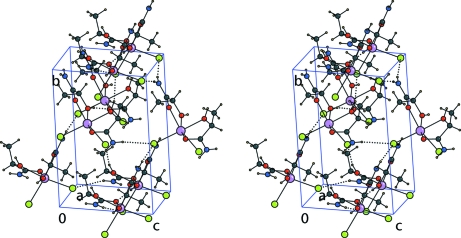
Stereoscopic view of molecular the stacking including hydrogen bonds.

## supplementary crystallographic information

### Comment

Metal-containing proteins have been studied in relation to severe diseases. Alzheimer's and mad cow diseases imply metal-protein interactions and metalloproteases are implied in cancer dispersion and angiotensin converting enzyme (ACE) in blood pressure control. For these reasons, amide-metal complexes have attracted much interest (lactamide = 2-hydroxypropionamide). Recently, we have been engaged in the synthesis and structural characterization of the cationic complexes [Zn(lactamide)_3_]^2+^ (Bekaert *et al.*, 2005) and [*B*(lactamide)_2_]^+^ (Bekaert *et al.*, 2007). Moreover, manganese is known to participate in a variety of biological reactions. We therefore studied and now report a new dichloromanganese(II) complex with lactamide ligands. Compound (1) (Fig. 1) contains one monomeric octahedral manganese complex, [Mn(C_3_H_7_NO_2_)Cl_2_]. Manganese is surrounded by two bidentate lactamide ligands each coordinating *via* the carbonyl O atom O1 (or O6) and the hydroxy atom O2 (or O7) and two Cl ligands. The complex exhibits a highly distorted octahedral geometry around the Mn^II^ ion with the apical positions occupied by O2 and Cl2 aoms (O2—Mn—Cl2: 167.63 (4) °). The Mn atom lies 0.270 (1) Å out of the basal plane (Cl1/O1/O2/O6). The degree of deviation from an ideal octahedron is appreciable, with the *cis* angles of the octahedron ranging from 72.33 (5) to 100.17 (6) °. The two equatorial Mn—O bond lengths for oxygen amide atoms are 2.196 (1) and 2.185 (2) Å for O1 and O6, respectively, being close to those reported for [Mn(*O*-acrylamide)_4_Cl_2_] with a similar coordination of Mn^II^ [2.186 (1) Å] (Girma *et al.*, 2005). Among the Mn—O (hydroxy) distances the equatorial Mn—O7 [2.174 (2) Å], is close to precedent values but very different from the axial Mn—O2 bond length [2.247 (2) Å]. The Mn—Cl bond distances [2.4535 (7) and 2.4786 (7) Å] are in good agreement with those found in similar octahedral Mn^II^ complexes like *e.g.* in [chloridobis(1,10- phenanthroline)(trichloroacetato)manganese(II)] [2.4374 (2) Å] (Chen *et al.*, 2006). Among the two possible coordination modes (N,*O* or O,*O*) in metal complexes with lactamide or its derivatives described in the literature, the title compound presents the O,*O* mode as in the before cited [Zn(lactamide)_3_]^2+^ and [*B*(lactamide)_2_]^+^ complexes. The packing is charaterized by numerous interactions that can be considered as hydrogen bonds since they correspond to H–A contacts significantly shorter than the sum of the van der Waals radii (Table 1). In particular, [Mn(lactamide)_2_Cl_2_] complexes are connected by N—H···Cl, N—H···O and O—H···O hydrogen bonds, generating a three dimensional network (Figures 1 and 2).

### Experimental

Enantiomerically pure (*S*)-lactamide ((*S*)-2-hydroxypropionamide, 2.22 g, 25 mmol) is dissolved in 20 ml of hot ethanoic acid and manganese(II) dichloride (1.26 g, 10 mmol) is added to this solution. The solution is kept at room temperature for 72 h. Pink crystals of the title compound slowly appear in the solution, whereupon crystals suitable for X-ray diffraction were obtained.

### Refinement

H atoms except those bonded to methyl groups were located in a difference map and refined and a common displacement parameter. For methyl groups H atoms were positioned geometrically and refined using a riding model, with C—H = 0.96Å with *U*_iso_(H) = 1.5 times *U*_eq_(C).

### Crystal data

**Table:**

[MnCl_2_(C_3_H_7_NO_2_)_2_]	*F*_000_ = 310
*M**_r_* = 304.03	*D*_x_ = 1.674 Mg m^−^^3^
Monoclinic, *P*2_1_	Mo *K*α radiation λ = 0.71073 Å
Hall symbol: P 2yb	Cell parameters from 25 reflections
*a* = 6.312 (2) Å	θ = 3.0–8.9º
*b* = 11.718 (3) Å	µ = 1.53 mm^−^^1^
*c* = 8.268 (2) Å	*T* = 293 (2) K
β = 99.47 (1)º	Parallelepiped, pink
*V* = 603.2 (3) Å^3^	0.18 × 0.16 × 0.12 mm
*Z* = 2	

### Data collection

**Table:**

Enraf–Nonius CAD-4 diffractometer	*R*_int_ = 0.030
Radiation source: fine-focus sealed tube	θ_max_ = 30.0º
Monochromator: graphite	θ_min_ = 2.5º
*T* = 293(2) K	*h* = −8→8
ω–2θ scans	*k* = 0→16
Absorption correction: none	*l* = −11→11
3659 measured reflections	3 standard reflections
1836 independent reflections	every 60 min
1803 reflections with *I* > 2σ(*I*)	intensity decay: none

### Refinement

**Table:**

Refinement on *F*^2^	Secondary atom site location: difference Fourier map
Least-squares matrix: full	Hydrogen site location: inferred from neighbouring sites
*R*[*F*^2^ > 2σ(*F*^2^)] = 0.022	H atoms treated by a mixture of independent and constrained refinement
*wR*(*F*^2^) = 0.061	*w* = 1/[σ^2^(*F*_o_^2^) + (0.0412*P*)^2^] where *P* = (*F*_o_^2^ + 2*F*_c_^2^)/3
*S* = 1.11	(Δ/σ)_max_ < 0.001
1836 reflections	Δρ_max_ = 0.35 e Å^−^^3^
161 parameters	Δρ_min_ = −0.51 e Å^−^^3^
1 restraint	Extinction correction: none
Primary atom site location: structure-invariant direct methods	

### Special details

**Table:**

Geometry. All e.s.d.'s (except the e.s.d. in the dihedral angle between two l.s. planes) are estimated using the full covariance matrix. The cell e.s.d.'s are taken into account individually in the estimation of e.s.d.'s in distances, angles and torsion angles; correlations between e.s.d.'s in cell parameters are only used when they are defined by crystal symmetry. An approximate (isotropic) treatment of cell e.s.d.'s is used for estimating e.s.d.'s involving l.s. planes.
Refinement. Refinement of *F*^2^ against ALL reflections. The weighted *R*-factor *wR* and goodness of fit *S* are based on *F*^2^, conventional *R*-factors *R* are based on *F*, with *F* set to zero for negative *F*^2^. The threshold expression of *F*^2^ > σ(*F*^2^) is used only for calculating *R*-factors(gt) *etc*. and is not relevant to the choice of reflections for refinement. *R*-factors based on *F*^2^ are statistically about twice as large as those based on *F*, and *R*- factors based on ALL data will be even larger.

### Fractional atomic coordinates and isotropic or equivalent isotropic displacement parameters (Å^2^)

**Table:**

	*x*	*y*	*z*	*U*_iso_*/*U*_eq_	
Mn1	0.28185 (4)	0.57573 (2)	0.28850 (3)	0.02305 (8)	
Cl1	0.43848 (9)	0.40435 (4)	0.43841 (7)	0.03790 (12)	
Cl2	0.13344 (8)	0.49119 (5)	0.02244 (5)	0.03331 (11)	
O1	−0.0161 (2)	0.56907 (16)	0.39299 (14)	0.0286 (3)	
N1	−0.1127 (4)	0.5206 (3)	0.6322 (2)	0.0513 (7)	
H1A	−0.228 (6)	0.480 (3)	0.576 (4)	0.041 (3)*	
H1B	−0.079 (6)	0.526 (3)	0.729 (4)	0.041 (3)*	
C1	0.0131 (3)	0.5736 (2)	0.54632 (19)	0.0287 (3)	
O2	0.3440 (2)	0.66403 (14)	0.53298 (18)	0.0333 (3)	
H2	0.406 (6)	0.730 (3)	0.563 (4)	0.041 (3)*	
C2	0.1925 (3)	0.6459 (2)	0.6381 (2)	0.0310 (4)	
H20	0.253 (5)	0.609 (3)	0.728 (4)	0.041 (3)*	
C3	0.1005 (7)	0.7558 (4)	0.6897 (6)	0.0699 (11)	
H3A	0.2141	0.8023	0.7469	0.105*	
H3B	−0.0016	0.7396	0.7607	0.105*	
H3C	0.0304	0.7958	0.5944	0.105*	
O6	0.2428 (2)	0.74635 (14)	0.1810 (2)	0.0332 (3)	
O7	0.5940 (2)	0.63077 (16)	0.2395 (2)	0.0414 (4)	
H7	0.726 (6)	0.617 (3)	0.278 (4)	0.041 (3)*	
N6	0.3807 (4)	0.89164 (17)	0.0554 (3)	0.0392 (4)	
H6A	0.483 (6)	0.919 (4)	0.025 (4)	0.041 (3)*	
H6B	0.255 (6)	0.924 (4)	0.036 (4)	0.041 (3)*	
C6	0.3966 (3)	0.79133 (17)	0.1305 (2)	0.0277 (3)	
C7	0.6136 (3)	0.73241 (18)	0.1480 (2)	0.0297 (4)	
H70	0.715 (6)	0.786 (4)	0.211 (4)	0.041 (3)*	
C8	0.6730 (4)	0.7018 (2)	−0.0168 (3)	0.0399 (5)	
H8A	0.8105	0.6647	−0.0006	0.060*	
H8B	0.6795	0.7700	−0.0802	0.060*	
H8C	0.5665	0.6513	−0.0741	0.060*	

### Atomic displacement parameters (Å^2^)

**Table:**

	*U*^11^	*U*^22^	*U*^33^	*U*^12^	*U*^13^	*U*^23^
Mn1	0.02205 (12)	0.02285 (12)	0.02441 (12)	0.00065 (10)	0.00426 (8)	0.00154 (9)
Cl1	0.0368 (2)	0.0242 (2)	0.0493 (3)	0.00502 (18)	−0.0032 (2)	0.00568 (18)
Cl2	0.0347 (2)	0.0378 (2)	0.02582 (17)	0.00258 (19)	0.00027 (15)	−0.00370 (18)
O1	0.0222 (5)	0.0419 (7)	0.0209 (5)	−0.0006 (6)	0.0014 (4)	0.0020 (6)
N1	0.0488 (11)	0.0796 (18)	0.0257 (7)	−0.0334 (12)	0.0060 (7)	0.0035 (9)
C1	0.0253 (7)	0.0362 (9)	0.0238 (7)	−0.0043 (9)	0.0018 (6)	0.0018 (8)
O2	0.0348 (7)	0.0298 (7)	0.0379 (7)	−0.0101 (6)	0.0134 (6)	−0.0101 (6)
C2	0.0313 (8)	0.0385 (9)	0.0225 (6)	−0.0089 (8)	0.0026 (6)	−0.0010 (7)
C3	0.070 (2)	0.0620 (19)	0.089 (2)	−0.0148 (18)	0.046 (2)	−0.0384 (19)
O6	0.0284 (6)	0.0280 (6)	0.0442 (7)	0.0033 (6)	0.0094 (6)	0.0096 (6)
O7	0.0202 (6)	0.0455 (9)	0.0578 (9)	0.0047 (6)	0.0043 (6)	0.0309 (8)
N6	0.0410 (10)	0.0281 (8)	0.0509 (10)	0.0063 (8)	0.0147 (8)	0.0128 (8)
C6	0.0295 (8)	0.0233 (8)	0.0299 (7)	0.0013 (6)	0.0041 (7)	0.0022 (6)
C7	0.0240 (7)	0.0279 (9)	0.0363 (8)	−0.0020 (7)	0.0020 (7)	0.0081 (7)
C8	0.0421 (11)	0.0343 (10)	0.0466 (11)	0.0121 (9)	0.0172 (9)	0.0065 (9)

### Geometric parameters (Å, °)

**Table:**

Mn1—O7	2.1739 (16)	C3—H3A	0.9600
Mn1—O6	2.1853 (17)	C3—H3B	0.9600
Mn1—O1	2.1964 (14)	C3—H3C	0.9600
Mn1—O2	2.2471 (15)	O6—C6	1.236 (3)
Mn1—Cl2	2.4535 (7)	O7—C7	1.427 (2)
Mn1—Cl1	2.4786 (7)	O7—H7	0.86 (4)
O1—C1	1.252 (2)	N6—C6	1.326 (3)
N1—C1	1.307 (3)	N6—H6A	0.80 (4)
N1—H1A	0.92 (4)	N6—H6B	0.87 (4)
N1—H1B	0.80 (3)	C6—C7	1.519 (3)
C1—C2	1.515 (3)	C7—C8	1.514 (3)
O2—C2	1.410 (2)	C7—H70	0.98 (4)
O2—H2	0.89 (4)	C8—H8A	0.9600
C2—C3	1.503 (4)	C8—H8B	0.9600
C2—H20	0.89 (3)	C8—H8C	0.9600
			
O7—Mn1—O6	72.40 (6)	C1—C2—H20	109 (2)
O7—Mn1—O1	160.90 (7)	C2—C3—H3A	109.5
O6—Mn1—O1	98.39 (6)	C2—C3—H3B	109.5
O7—Mn1—O2	90.10 (7)	H3A—C3—H3B	109.5
O6—Mn1—O2	86.32 (7)	C2—C3—H3C	109.5
O1—Mn1—O2	72.33 (5)	H3A—C3—H3C	109.5
O7—Mn1—Cl2	100.17 (6)	H3B—C3—H3C	109.5
O6—Mn1—Cl2	90.23 (5)	C6—O6—Mn1	119.04 (14)
O1—Mn1—Cl2	96.49 (4)	C7—O7—Mn1	120.43 (12)
O2—Mn1—Cl2	167.63 (4)	C7—O7—H7	101 (2)
O7—Mn1—Cl1	91.97 (5)	Mn1—O7—H7	137 (2)
O6—Mn1—Cl1	162.47 (5)	C6—N6—H6A	120 (3)
O1—Mn1—Cl1	94.10 (5)	C6—N6—H6B	118 (3)
O2—Mn1—Cl1	85.82 (5)	H6A—N6—H6B	122 (4)
Cl2—Mn1—Cl1	100.56 (3)	O6—C6—N6	122.2 (2)
C1—O1—Mn1	113.83 (11)	O6—C6—C7	121.32 (18)
C1—N1—H1A	118 (2)	N6—C6—C7	116.43 (19)
C1—N1—H1B	115 (3)	O7—C7—C8	109.57 (18)
H1A—N1—H1B	127 (3)	O7—C7—C6	105.93 (15)
O1—C1—N1	121.9 (2)	C8—C7—C6	112.02 (17)
O1—C1—C2	120.30 (17)	O7—C7—H70	111 (2)
N1—C1—C2	117.71 (16)	C8—C7—H70	113.3 (18)
C2—O2—Mn1	116.74 (12)	C6—C7—H70	105 (2)
C2—O2—H2	106 (2)	C7—C8—H8A	109.5
Mn1—O2—H2	131 (2)	C7—C8—H8B	109.5
O2—C2—C3	112.3 (2)	H8A—C8—H8B	109.5
O2—C2—C1	107.58 (15)	C7—C8—H8C	109.5
C3—C2—C1	109.2 (2)	H8A—C8—H8C	109.5
O2—C2—H20	110 (2)	H8B—C8—H8C	109.5
C3—C2—H20	108 (2)		

### Hydrogen-bond geometry (Å, °)

**Table:**

*D*—H···*A*	*D*—H	H···*A*	*D*···*A*	*D*—H···*A*
N1—H1A···Cl1^i^	0.92 (4)	2.40 (4)	3.310 (2)	170 (3)
N1—H1B···Cl2^ii^	0.80 (3)	2.60 (4)	3.360 (2)	159 (3)
O2—H2···Cl1^iii^	0.89 (4)	2.26 (4)	3.1250 (17)	164 (3)
O7—H7···O1^iv^	0.86 (4)	1.83 (4)	2.676 (2)	167 (3)
N6—H6A···Cl2^v^	0.80 (4)	2.65 (4)	3.439 (2)	168 (3)
N6—H6B···Cl2^vi^	0.87 (4)	2.54 (4)	3.409 (3)	172 (4)

Symmetry codes: (i) *x*−1, *y*, *z*; (ii) *x*, *y*, *z*+1; (iii) −*x*+1, *y*+1/2, −*z*+1; (iv) *x*+1, *y*, *z*; (v) −*x*+1, *y*+1/2, −*z*; (vi) −*x*, *y*+1/2, −*z*.
